# A Bio-Based Resin for a Multi-Scale Optical 3D Printing

**DOI:** 10.1038/s41598-020-66618-1

**Published:** 2020-06-16

**Authors:** Edvinas Skliutas, Migle Lebedevaite, Sigita Kasetaite, Sima Rekštytė, Saulius Lileikis, Jolita Ostrauskaite, Mangirdas Malinauskas

**Affiliations:** 1 Laser Research Center, Physics Faculty, Vilnius University, Sauletekio Ave. 10, Vilnius, LT-10223 Lithuania; 2 Department of Polymer Chemistry and Technology, Kaunas University of Technology, Radvilenu Rd. 19, LT-50254 Kaunas, Lithuania; 3 3D Creative Ltd., Mokslininku St. 2a, Vilnius, LT-08412 Lithuania

**Keywords:** Ecology, Chemical engineering, Techniques and instrumentation, Other photonics

## Abstract

Materials obtained from renewable sources are emerging to replace the starting materials of petroleum-derived plastics. They offer easy processing, fulfill technological, functional and durability requirements at the same time ensuring increased bio-compatibility, recycling, and eventually lower cost. On the other hand, optical 3D printing (O3DP) is a rapid prototyping tool (and an additive manufacturing technique) being developed as a choice for efficient and low waste production method, yet currently associated with mainly petroleum-derived resins. Here we employ a single bio-based resin derived from soy beans, suitable for O3DP in the scales from nano- to macro-dimensions, which can be processed even without the addition of photoinitiator. The approach is validated using both state-of-the art laser nanolithography setup as well as a widespread table-top 3D printer - sub-micrometer accuracy 3D objects are fabricated reproducibly. Additionally, chess-like figures are made in an industrial line commercially delivering small batch production services. Such concept is believed to make a breakthrough in rapid prototyping by switching the focus of O3DP to bio-based resins instead of being restricted to conventional petroleum-derived photopolymers.

## Introduction

Bio-based polymers are emerging as replacement for petroleum-derived polymers. The growth of the production and market is 2.05 Mtons of bio-plastics^1^ and 700 bilion Euros in Europe only^2^. The main advantages of bio-based plastic products compared to the conventional plastics are the preservation of fossil resources by using bio-mass which is a renewable resource and provision of the unique potential of carbon neutrality, as well as bio-degradability of the certain types of bio-plastics which offers additional means of recovery at the end of a product’s life^3^. The spectrum of bio-based plastics usage varies from nanocomposites^4–8^ and films^9–11^ to adsorbents^12–14^. Vegetable oils are potential starting materials for the preparation of polymers due to their ready availability, inherent bio-degradability, negligible toxicity, and existence of modifiable functional groups^15^. Nowadays there are a lot of scientific research dedicated to the light induced polymerization. As there exist diverse technical implementations of this technology, it is known in many names: lithography (stereolithography, digital light processing (DLP)/projection lithography), direct laser writing (DLW) or alternatively laser direct writing (LDW), two-photon polymerization (2PP), nonlinear lithography (NLL), multi-photon lithography (MPL), etc. However, this additive manufacturing process simply can be called by one common name: optical 3D printing (O3DP) as it is based on photons. This rapid prototyping tool is being developed as a choice for efficient and low waste production tool, yet currently associated with mainly petroleum-derived resins^16–19^. On the other hand, O3DP in combination with post-processing (thermal-treatment) allows fabrication of free-form structures which can serve as 3D templates for realization of pure glass^20^, ceramic^21^, metal^22^ and crystalline^23^ objects. Additionally, O3DP offers flexible manufacturing of multi-scale (multi-dimensional) hierarchical^24^ or arbitrary^25^ structures which allows speeding up^26^ the printing yet keeping the nano-/micro-functionalities available in macro-dimensional objects suitable for diverse practical applications^27^. Even graytone^28^, multi-material^29^, selectively erasable^30^ and hybrid subtractive-additive-merging^31^ light assisted 3D manufacturing was demonstrated recently showing its unrestricted potential in advanced material engineering, rapid prototyping and flexible production.

Here we employ a single bio-based resin derived from soy beans, suitable for O3DP in the scales from nano- (hundreds nm) to macro-dimensions (cm). Furthermore, optimizing the pulsed-exposure the resin can be processed pure (without the addition of any photoinitiator (PI)). The approach is validated using both state-of-the art laser nanolithography setup^32^ as well as a widespread table-top 3D printer^33^ - sub-micrometer accuracy features and macro-scale 3D objects are fabricated reproducibly. Additionally, chess-like figures (“Marvin” and “Tower”) are made in an industrial line (standard Formlabs Form 2 optical 3D printer using default settings) proving its suitability for commercially delivering small batch production services on demand^34^. Such proposed concept is experimentally validated and characterized and is believed to make an immediate breakthrough in rapid prototyping by switching the focus of O3DP to bio-based resins instead of conventional petroleum-derived photopolymers.

## Results

### Material characterization

**Figure 1:**
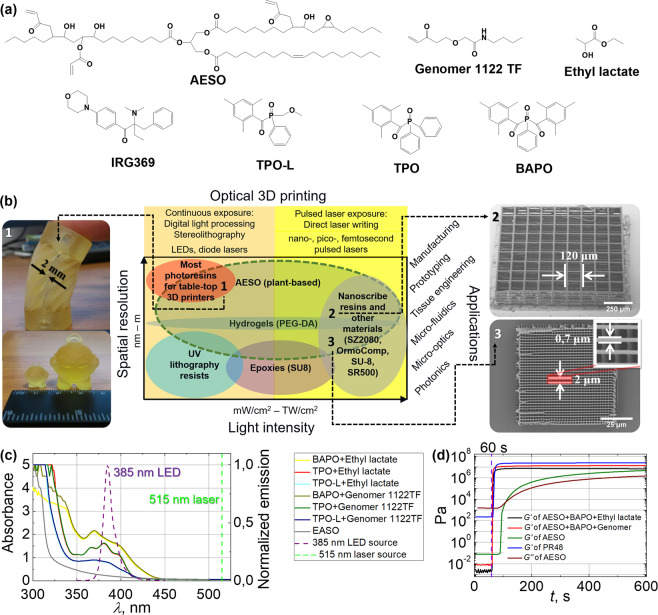
Potential applicability and characteristics of AESO. (**a**) – chemical structures of AESO, diluents Genomer 1122TF and ethyl lactate, PIs IRG369, TPO-L, TPO and BAPO. (**b**) – an explanation chart for the materials used in optical 3D lithography: Y-left axis – achievable spatial resolution, Y-right – available applications, X-top – required equipment, X-bottom – required irradiation intensities. Images of the objects produced out of AESO-based resin using both DLP and NLL technologies are shown. The numbers 1–3 are marked on the chart to represent how the objects were produced and indicate their potential applications. (**c**) – measured AESO, AESO + PI(1% w/w)+diluent absorbance and normalized light source emission spectra. Green dashed vertical line marks the wavelength of the laser source. (**d**) – the dependencies of storage modulus *G*’ of AESO, AESO-based resins, and PR48 on irradiation time^43,64^. The onset of irradiation (60 s) is marked with violet vertical dashed line.

Acrylated epoxidized soy bean oil (AESO) was chosen to demonstrate bio-based resins’ applicability in O3DP, as primary experiments were shown in S. Miao paper^35^, but expanding to the detailed substance examination for photopolymerization including both digital light processing (DLP) lithography and nonlinear laser lithography (NLL). Chemical formulations of used ingredients are presented in Fig. 1(a). DLP and NLL are two additive manufacturing techniques allowing the production of the diverse objects out of photosensitive resins through polymerization reaction, induced via linear (DLP) or nonlinear (NLL) light-matter interaction. An explanation chart in the Fig. 1(b) represents materials used in the O3DP and their suitability for certain technological implementations, applications, required irradiation intensities and achievable resolution. For example, most of the table-top 3D printers are compatible with acrylate or (meth)acrylate based resins for the prototyping of macro-scale objects. UV lithography resins or epoxies can be used for the fabrication of micro-(/nano-)scale 2D structures using appropriate equipment. Hydrogels such as bio-degradable poly(ethylene glycol) diacrylate (PEG-DA) are applicable in tissue engineering creating 3D, hydrated, bio-mimetic structures for the encapsulation of living cells^36^. On the other hand, PEG-DA was demonstrated independently to be suitable with both technical implementations: DLP and NLL^37^. There exist photoresins typically used only for the high intensity (TW/cm^2^) NLL-dedicated manufacturing of objects at nano-scale. For example, it is OrmoComp, SU-8, SCR500, hybrid materials as SZ2080 and etc. On the other hand, although Nanoscribe GmbH provides multi-scale 2PP, it actually use three different photoresins specifically distinct for each spatial-scale (mm, *μ*m and nm), meaning an absence of the single photoresin, suitable for really scalable manufacturing. Lastly, all of them are petroleum-derived, but not derived from renewable-resources. As compared with common O3DP materials, AESO has the advantage of being suitable for manufacturing objects that span five orders of magnitude. Although compatibility of PEG-DA with both DLP and NLL was reported in separate studies, there are only directly non-related publications showing such applicabilities. On the contrary, we emphasize AESO multi-scale printing in a single experimental work. Not only presence or absence of light absorbing compounds such as photoinitiators (PIs) makes resins suitable for one or other technique, but the final chemical formulation, physical characteristrics as viscosity and diffusion as well as reaction mechanisms has significant impact determining the 3D photostructuring, its quality and repeatability. Currently there is no report showing the exact resin formulation can be suitable for continuous spatial scaling applying several lithography techniques. The produced bench-marking samples are shown in the numbered insets at the sides of the chart, representing structures in different scales: by DLP layer-by-layer fashion produced cm sized “Tower” and “Marvin” (1) and by NLL manufactured scaffold structure (2) and grating (3) reaching up to hundreds *μ*m of external dimensions and only few *μ*m of individual features. These objects are tagged in the chart (numbers in black) according to the used technology, achieved scale and potential applications.

Measured absorbance spectra of non-photosensitized and diluent free AESO and AESO mixed with 1% w/w PI dissolved in a diluent (ethyl lactate or Genomer 1122TF) are demonstrated in Fig. 1(c). Photosensitized AESO spectra showed a good overlap with employed LED source emission spectrum. Taking into account only the central wavelength (385 nm) of the light source, absorbance *A*, absorption coefficient α (derived from *A*, see Equation S1.1) and light penetration depth *h*_*a*_ (*h*_*a*_ = *α*^−1^) were evaluated. AESO without PI and diluent is a low absorbent material for the used irradiation. *α* values were about 5.1 cm^−1^ resulting in high penetration depth – 2 mm. Such parameters are inconvenient for the layered fabrication manner of O3DP, because of resolution loss in Z axis. Also, no polymerization was observed exposing AESO with 385 nm wavelength light source. PI addition increased *A* by one order. The most absorbent PI was BAPO as *h*_*a*_ was reduced to 250 *μ*m for 385 nm irradiation (see Table 1). Resin with TPO showed lower absorption: as compared with BAPO, the calculated *h*_*a*_ was 1.2 times higher and reached 300 *μ*m. The least impact had TPO-L PI as *h*_*a*_ was decreased approximately only to 550 *μ*m. Obtained *h*_*a*_ values in AESO mixed with PIs are in fine agreement with the ones of the commercial materials, which mostly vary from dozens to several hundreds *μ*m: PR48 – 80 *μ*m, PlasClear – 120 *μ*m, FSL Clear 320 *μ*m^38^. Diluents did not have a noticeable impact on resins’ absorbance spectra. As can be seen from the spectra, the applied laser source irradiation at 515 nm wavelength can be absorbed nonlinearly, which would correspond to the linear ultra-violet (UV) light absorption of twice shorter wavelength (257 nm). However, in this case only tightly focused irradiation, whose intensity (*I*) exceeds polymerization threshold, can be taken into account. Polymerization threshold defines a minimal amount of *I* required to induce the non-reversible polymerization (cross-linking) reaction. This in contrast to the linear absorption mechanism, where the main role is defined via absorbed energy dose *D* (directly proportional to *I* and duration the irradiation was applied (Supplement Eq. S2.2)). Usually, the polymerization threshold is exceeded in a confined space where the light is being focused, defining a volumetric pixel – voxel. Thus it enables manufacturing of 3D objects at *μ*m scale.

**Table 1:** AESO and its based resins optical, rheological and bio-renewable carbon content characteristics.

Resin	*A*	*α* [cm^−1^]	*h*_*a*_ [*μ*m]	*G*’ [MPa]	*G*” [MPa]	*t*_*gel*_ [s]	*BRC%*
AESO	0.22	5.1	1970	4.8^43^	1.5^43^	49^43^	86
AESO + BAPO + ethyl lactate	1.73	39.9	250	6.5	0.5	3.6	89
AESO + BAPO + Genomer 1122TF	1.77	40.8	245	14.2	6.2	3.7	64

Evolution of AESO, AESO-based resins, and PR48 (which formulation includes same TPO PI and Genomer 1122TF diluent^39^) rheological characteristics obtained from real-time photorheometry are presented in Fig. 1(d). After starting the exposure with 250–450 nm light, the values of storage modulus *G*’ and loss modulus *G*” (depicted only for AESO) started to increase. It indicated growth of chains and three-dimentional polymer network formation in the initial stage of photocross-linking. Continuing the process, *G*’ was increasing faster than *G*” and finally exceeded it. The point where *G*’ = *G*” defines the gel point *t*_*gel*_, when a high viscosity Newtonian fluid begins to transform into a hard elastic polymer. At this point the ratio of the viscous and the elastic portion of the viscoelastic deformation behaviour (*G*”/*G*’) starts to decrease (depicted in Supplementary Fig. S1.1). The ratio is known as a loss factor *tan δ* and it decreases further until the final degree of cross-linking is reached. As the resin doped with BAPO demonstrated the most perspective optical characteristics, its rheological properties were measured and compared to the non-photosensitized AESO. Exact values of *G*’, *G*” and *t*_*gel*_ are shown in Table 1. Diluents had a great impact on the resins rheological properties and the rate of photocross-linking. Resins with diluents and PI indicated significantly quicker photocross-linking process when prompt increment of *G*’ values after the onset of irradiation was monitored. Using ethyl lactate, *G*’ was increased 1.4 times (6.5 MPa), however *G*” was reduced 3 times (0.5 MPa). Such changes led to the polymerized films being soft and likely less practically applicable in the layered manufacturing. Genomer 1122TF helped to increase *G*’ about 3 times (14.2 MPa) and *G*” 4 times (6.2 MPa), resulting in a thoroughly cross-linked polymer network and rigid polymerized films. These values are the most matching with the ones, acquired when measuring commercial resins: PR48 – *G*’ = 26 MPa, *G*” = 8.4 MPa, PlasGray – *G*’ = 30 MPa, *G*” = 9.3 MPa, confirming AESO as an appropriate replacement for synthetic (meth)acrylates. Thus the following investigation of AESO suitability for DLP lithography was proceeded with Genomer 1122TF diluent. Due to PI, *t*_*gel*_ was decreased about 13 times. It means that the resin can be polymerized after exposure of a few seconds duration, which is desired for applications in O3DP.

Bio-renewable carbon (*BRC*) content is calculated according to the Supplement Eq. S1.1. Its values for AESO and AESO-based resins are depicted in Table 1 and represent the percentage of carbon, derived from bio-renewable resources. Which is in all cases above 50% and might reach more than 80% in applied resin formulations. The mechanical properties (elastic modulus, tensile strength and elongation at break) of photocross-linked AESO resins are being investigated and will be published in a separate article (M. Lebedevaite *et al*.) comparing with J. Guit *et al*. provided mechanical performance of their self synthesized epoxidized soybean oil^40^.

### Digital light processing lithography

**Figure 2:**
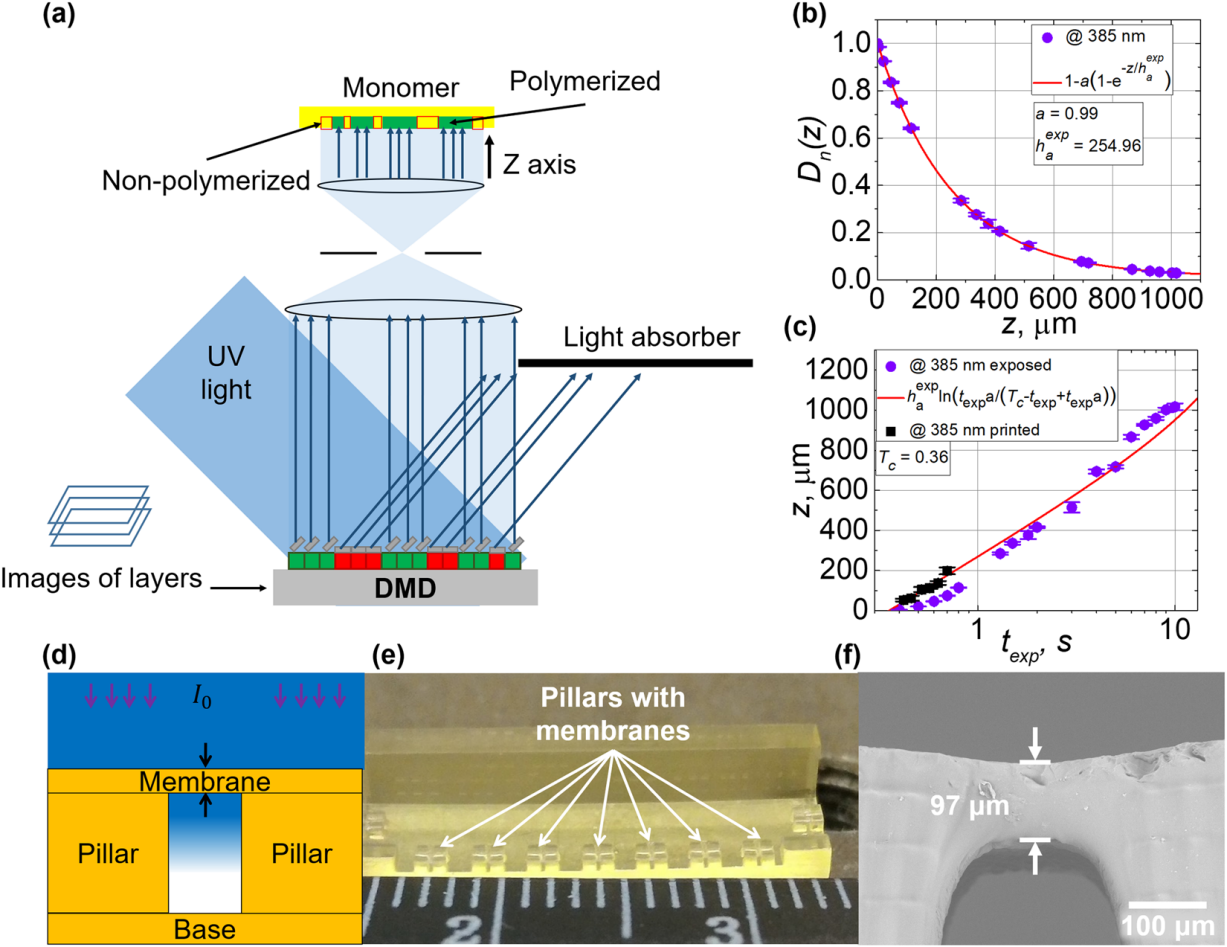
Assessment of the exposure conditions for the DLP lithography and obtained results. (**a**) – a scheme of the DLP lithography. DLP experiments data using AESO + BAPO + Genomer 1122TF resin. (**b**) – normalized energy dose *D*_*p*_ dependence on polymerized films height *z*. (**c**) – polymerized films height *z* dependence on exposure duration *t*_*exp*_. (**d**) – a model of a single layer membrane on the pillars. (**e**) – the model produced out of AESO based resins. (**f**) – printed membrane: theoretical height 102 *μ*m, measured 97 *μ*m (SEM image). Values of the measured thicknesses of the printed membranes (five black squares) are shown in picture (**c**).

Next AESO-based resins (doped with 1% w/w PI and diluted with Genomer 1122TF, which consisted 1/3 of the monomer mass) were polymerized varying irradiation exposure duration *t*_*exp*_ from 0.1 to 10 seconds, which enabled to accurately determine a light penetration depth for a resin ${h}_{a}^{{\exp }}$ and a critical exposure duration *T*_*c*_ from obtained different thickness films. ${h}_{a}^{{\exp }}$ actually represents the same parameter *h*_*a*_ as mentioned in Section 2.1, but is marked with index *exp* to distinguish the method it was calculated. *T*_*c*_ defines the minimal exposure duration, required to polymerize the resin with given light source, producing an intensity *I*. All the experiments described in this Section were performed with Asiga Pico2 39 UV optical 3D printer. A principal scheme of the DLP lithography is depicted in the Fig. 2(a). Fixed amount of the resin was exposed to the 385 nm wavelength irradiation (by 2 mm radius spots and *I* = 30 mW/cm^2^ according to readings on the device screen). Then the heights of the polymerized films were measured using optical profilometer. Following ${h}_{a}^{{\exp }}$ and *T*_*c*_ calculations were based on Beer-Lambert law mathematical method, described in H. Gong’s papers^38,41^ and Supplementary document. At first, normalized exposure dose *D*_*n*_*(z)* values were calculated and plotted as a function of polymerization thickness *z* (see Fig. 2(b)). Approximation with Eq. S1.4 revealed ${h}_{a}^{{\exp }}$ and *a* parameters. *a* parameter describes a spectral overlap between spectra of material absorbance and light source emission and its value was 0.99 for 385 nm wavelength. The determined ${h}_{a}^{{\exp }}$ parameter for 385 nm light was 255 *μ*m, which is in reasonable agreement with values of *h*_*a*_ achieved from absorbance spectra. *T*_*c*_ was assessed from the *z* dependence on *t*_*exp*_ (Fig. 2(c)), applying approximation of the experimental data with Eq. S1.5, when ${h}_{a}^{{\exp }}$ and *a* values were fixed as calculated from previous graph. It gave the result of *T*_*c*_ = 0.36 s. Additionally AESO-based resins were prepared with various concentrations of BAPO (0.25, 0.5, 1, and 2% w/w). By varying the amount of BAPO, the possibility to control ${h}_{a}^{{\exp }}$ and *T*_*c*_ was demonstrated: it was reduced from 700 *μ*m to 200 *μ*m and from 1 s to 0.15 s, by increasing the ratio of the PI in the mixture.

From the obtained parameters, the polymerization depth *z*_*p*_ was calculated for numerous *t*_*exp*_ for the resin with 1% w/w amount of PI. Then the monolayer membranes on supportive pillars were printed under the selected five different exposure doses (see Fig. 2(d) for the scheme and (e) for the printed object). Membranes’ thicknesses were measured from SEM images (see Fig. 2(f)) and averaged of 6–13 samples. It allowed to evaluate if polymerized material thickness conforms with the calculations of *z*_*p*_. To visualize, how the obtained thicknesses of the polymerized membranes match with the calculations, the measured data was plotted along the approximation function used in Fig. 2(c) (marked in black squares). It showed, that experimental results were in good compliance with the applied theoretical Lambert-Beer model.

### Nonlinear laser lithography

**Figure 3:**
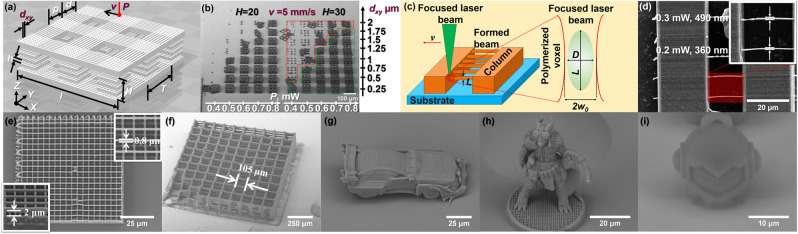
Manufacturing via NLL and obtained results. (**a**) – a model of 75 × 75 *μ*m^2^ size bi-layer scaffold structure: *T* – 30 *μ*m period, *p* – 15 *μ*m log width, *l* – 75 *μ*m log length, *d* – 15 *μ*m distance between logs, *d*_*xy*_ – distance between scans, *H* – 20 or 30 *μ*m vertical column height, *h* – 5 *μ*m height between separate column segments, *P* – applied laser power, *v* – scanning velocity. (**b**) – SEM image (at 45° angle) of arrays of manufactured scaffold structures out of AESO. Applied average *P* was 0.4–0.8 mW (recalculated to intensity *I* 1.2–2.4 mW/cm^2^) and is represented in white scale at the bottom. *d*_*xy*_ is shown on the black scale at right. *v* was set to 5 mm/s. The green area marks well-shaped objects and the red – deformed ones or only their residuals. (**c**) – representation of the “resolution bridges” (RB) method and ellipsoid shape voxel (green) at the 2w_0_ diameter laser beam focal plane. *D* and *L* are lateral and longitudinal dimensions of the voxel. (**d**) – demonstrates a measurement of RB lateral *D* size (top view). Applied *v* to form beams was set to 1 mm/s, *P* was altered in the range 0.1–0.6 mW. (**e**) – 2D grating in AESO + 0.5% w/w IRG369, *v* = 0.15 mm/s, *P* = 0.12 mW, *h* = 1 *μ*m. *D* and *L* sizes are represented in upper-right and lower-left insets, respectively. (**f**) – 1.065 × 1.065 mm^2^ size 7 layers scaffold: *p* = 25 *μ*m, *d* = 105 *μ*m, *v* = 5 mm/s, *P* = 0.6 mW; SEM images (at 45° angle) of objects fabricated using *P* = 0.18 mW: (**g**) – “Car” model: *v* = 1.8 mm/s, *d*_*xy*_ = 0.15 *μ*m; (**h**) – “Tower” model: *v* = 1.2 mm/s, *d*_*xy*_ = 0.15 *μ*m; (**i**) – “Marvin” model: *v* = 1.2 mm/s, *d*_*xy*_ = 0.25 *μ*m. For fabrication of the objects in pictures (**b**) and (**f**) 20 × 0.8 NA objective was used, for (**d**–**e**) and (**g**–**i**) – 63 × 1.4 NA.

In this part AESO suitability for NLL will be discussed. First of all, fabrication parameters were assessed, by manufacturing arrays of the 75 × 75 *μ*m^2^ size bi-layer scaffold structures, shown in Fig. 3(a). Average laser power (*P*) was varied in the range of 0.1–1.2 mW, scanning velocity *v* = 2.5–15 mm/s, and distance between adjacent linear scans *d*_*xy*_ = 0.25–2 *μ*m. *P* and *v* parameters define the exposure conditions, corresponding to the voxel size at the focus plane. According to selected *P* and *v*, appropriate *d*_*xy*_ must be selected so that adjacent scanned areas would overlap to create a solid line, but not to overexpose the material. Used objective was 20 × 0.8 NA, resins were non-photosensitized AESO and mixed with 1% w/w BAPO and ethyl lactate or Genomer diluents (1/3 of monomer mass). A SEM image of manufactured structures out of AESO is presented in Fig. 3(b). The main varied parameters are shown on the scales. By comparing the produced objects out of photosensitized resins, it was assessed, that when using ethyl lactate the structures with higher *d*_*xy*_ values (> 1.25 *μ*m) under *P* = 0.4–0.8 mW (*I* = 1.2–2.4 TW/cm^2^) irradiation were strongly deformed or even did not withstand the development process. Meanwhile when Genomer was used as a diluent the acquired samples showed better survivability under the same experimental conditions. It is related to the higher *G*’ and *G*” values, which make the polymerized structures more rigid. Increasing *P* helped to decrease the deformation of the objects and enhance their resistance to the development process. Unfortunately, samples with ethyl lactate were negatively affected by the increased *P* – substance was overexposed creating gas bubbles during fabrication and causing distortion of the scaffold structure. While manufacturing in the resins with PI was less dependant on the *P*, *v* and *d*_*xy*_ variation, PI-free AESO showed higher sensitivity to the adjustment of these parameters. Even a slight increment (0.25 *μ*m) of *d*_*xy*_ parameter, while rest of two were fixed, could lead to strongly deformed or at all not formed objects (Fig. 3(b)). Increase of the power *P* up to 0.9–1.2 mW allowed to manufacture objects applying higher *d*_*xy*_ values, resulting to enhanced throughput. Evaluated ranges of the *P*, *v*, and *d*_*xy*_ parameters when well-defined objects were obtained determines the fabrication window (FW) - a ratio of the optical damage and polymerization threshold. For AESO and AESO-based resins with PI and diluents, optimal values to produce scaffold structures in a reasonable time (a few minutes) were: *P* = 0.4–1.2 mW (*I* = 1.2–3.7 TW/cm^2^), using *v* = 2.5–10 mm/s and *d*_*xy*_ = 0.25–1.5 *μ*m. It corresponds to FW = 3, which is technically feasible. In case only the lower *v* can be applied, *P* is altered accordingly as shown in further examples. Also, the geometry of the object must be taken into account when talking about its manufacturability. For instance, scaffold structures with higher vertical columns (30 *μ*m) tend to be more deformed than the smaller ones (20 *μ*m), which is determined by the rheological properties of the material. Thus in order to produce certain objects out of AESO, both manufacturing parameters and geometry of the model must be well set.

To investigate, what are particular sizes of the features formed from a single tightly focused laser scan, a “resolution bridges” (RB) method was used (Fig. 3(c))^42^. Hanging beams between supportive columns were formed using previously assessed manufacturing parameters: *v* = 0.5 mm/s, *P* = 0.18 mW and *d*_*xy*_ = 0.25 *μ*m for the columns and for the beams – varying *v* in the range of 0.01–10 mm/s and *P* = 0.1–0.6 mW (*I* = 0.27–1.6 TW/cm^2^ using 63 × 1.4 NA objective). By measuring beams lateral *D* and longitudinal *L* dimensions, a voxel size can be approximately determined (for the exact explanation, please see Supplementary document). The SEM images of RB are shown in the Fig. 3(d). Most of the formed beams were uneven, bent, twisted, stretched if the supportive columns have moved apart, sometimes adjacent beams tend to merge. It had a great impact on the rise of deviation of the measured lateral *D* and longitudinal *L* sizes of the beams as some of them appeared bigger or smaller due to their twist in respect of the viewing point. Thus we were not able to determine a detailed *D* and *L* dependency on varied exposure parameters and only certain values were evaluated from smoothly formed beams. It was assessed that *D* size can reach several hundreds nm. The smallest achieved aspect ratio (*D*/*L*) was 1.5. Also, such deformations of the beams confirmed low storage modulus of the material (4.8 × 10^6^ Pa^43^), which has to be taken into account while modelling 3D structures’ geometry. Other examples of various polymerized objects are demonstrated in the SEM images in the Fig. 3(e–i). (e) shows 75 × 75 *μ*m^2^ 2D grating made in AESO + 0.5% w/w IRG369 with 63 × 1.4 NA objective. Applying *v* = 0.15 mm/s and *P* = 0.12 mW, well-defined grating having 0.8 *μ*m wide and 2 *μ*m high logs was manufactured. To show the ability to polymerize bulky objects having fine features, the models of “Car”, “Dragon” and “Marvin” were produced in non-photosensitized AESO (Fig. 3(g–i)). An example of 1.065 × 1.065 mm^2^ size scaffold produced out of non-photosensitized AESO with 20 × 0.8 NA objective is demonstrated in the Fig. 3(f). Objects of this type and size can be applied for tissue regeneration applications *in vitro* as well as *in vivo*^27^. All the objects were manufactured as they are, without the need of any supportive structures. Due to the fact that the material is relatively soft, some parts of the models were deformed. However, structures with fine 3D features were achieved.

## Discussion

Currently, optical 3D printing (O3DP) has become a precise additive manufacturing technology, enabling production of diverse objects out of photosensitive materials via light induced polymerization reaction. O3DP can be classified into two main groups, which are discerned by to the fundamentals of physics depending on radiation intensity and wavelength: light travelling through the substance can be absorbed linearly or nonlinearly. While the former ensures high throughput and rapid manufacturing of macro-scale objects, the latter allows high spatial resolution fabrication in micro-scale and true 3D structuring. To utilize the benefits of either one, various polymerization machines (table-top devices, custom made prototypes, state-of-the-art scientific and industrial setups) were developed. From the technical point of view, both (linearly and nonlinearly induced photopolymerization) are already well studied: achievable spatial resolution, manufacturing throughput and printable objects sizes. The applications part is well examined too, and covers a wide range of realizations: from rapid prototypes manufacturing^44^ and medicine^45^, to delicate sensors^46^, lab-on-chip^47^, metamaterials^48^ and micro-optics^49^ fabrication. Recently, a lot of attention has been focused to the materials used in O3DP for their functionality. There is a demand for new photosensitive resins that could fulfill requirements for certain applications: be resilient to the high temperature^21^, have tunable refractive index^50^, be bio-compatible^51^ or bio-derived materials^52,53^. Thus growing number of publications, investigating the ways how to examine the suitability of the resins for the particular technology and applications^41,54^, is appearing. Also, high interest is shown for the sustainable bio-based products having potential to reduce the adverse environmental impact and be eco-friendly^55–57^. In some cases there arises a need to merge pros of both linearly and nonlinearly induced photopolymerization (rapid manufacturing + high spatial resolution) for one material^25,58,59^. However, by this time there is no demonstration of a single resin suitable for the linear and the nonlinear O3DP, maintaining both high throughput and spatial resolution. Usually, the material is proper only for one technological fulfillment because of its properties, for example, optical characteristics, viscosity, impurities, sensitivity to development and post-processing, photopolymerization mechanism^60^, etc. In this paper, it was demonstrated that the investigated bio-based AESO was advantageous as compared to the existing known resins for the O3DP, in a sense that it can be processed with either DLP lithography, or NLL, maintaining over 60% of bio-renewable carbon. As rheological (storage modulus, loss modulus, viscosity) and optical (absorption) properties of AESO were easily modified by mixing it with diluents and PIs, we showed propriety of custom made resins for two technological implementations, enabling to produce multi-scale (from hundreds nm to cm) objects from a single material. This could be beneficial for science and industry as a two step technique ensuring high throughput and spatial resolution manufacturing. In comparison to previously reported achievements, where photostructuring of AESO via linear absorption employing pulsed laser radiation and a custom made setup was demonstrated^35^, our proposed approach revealed the ability to perform a 3D macro-scale rapid manufacturing of the same monomer employing light engine, based on LED radiation projection. We foresee this implementation to be radically simpler and more affordable for regular users of O3DP owning in the market available table-top 3D printers. A similar approaches were presented by two scientific groups, who has recently investigated a suitability of used cooking oil^61^ and their self synthesized via the methacrylation epoxidized soybean oil^40^ for commercial DLP 3D printers. Another uniqueness of our work is the demonstration of 3D structuring employing NLL with and without PI (on demand). Tightly focused femtosecond radiation allows non-linear absorption to occur, resulting in the production of free-standing micro-scale objects, despite the specific pre-polymer mixture. The throughput of manufacturing at high-spatial resolution can be increased by using a microtransfer molding technique^62^, which is suitable and for bio-based systems^63^, as it was shown recently. In general, AESO has a great potential to be straight-forwardly applied in a multi-scale fabrication, employing O3DP technologies, which is a naturally evolving development direction.

## Materials and Methods

### Materials

Custom made photosensitive resins were based on AESO (purchased from Sigma-Aldrich), which was mixed with three different PI: phenylbis(2,4,6-trimethylbenzoyl)phosphine oxide (BAPO, Sigma-Aldrich), diphenyl(2,4,6-trimethylbenzoyl)phosphine oxide (TPO, Rahn) and ethyl(2,4,6-trimethylbenzoyl)phenylphosphinate (TPO-L, Fluorochem). Two diluents to control resins viscosity were used: bio-degradable ethyl lactate (Sigma-Aldrich) and reactive diluent Genomer 1122TF (Rahn). Additionally, 2-benzyl-2-dimethylamino-1-(4-morpholinophenyl)-butanone-1 (IRG369, Sigma-Aldrich) PI was used for NLL experiments. Resins were prepared by mixing AESO with diluents (1/3 of AESO mass) along with variable amount of PI (0.25, 0.5, 1, 2% w/w of AESO mass). Compounds were stirred with magnetic stirrer overnight in amber glass bottles to protect them from the light. To achieve better mixing performance, the compounds were heated up to 35 °C. Compounds’ absorbance spectra were obtained using Shimadzu UVProbe spectrophotometer.

### Equipment

#### Digital light processing lithography

We used digital light processing (DLP) optical 3D printers Asiga Pico2 39 UV and Autodesk Ember to test custom made resins for linear absorption and suitability for macroscale additive manufacturing. Results obtained using Ember are not shown intentionally as they gave only supportive and no additional specific data. Technical specifications of Asiga Pico2 39 UV can be found in Supplementary Table S2.1. Light engine was based on a Texas Instruments (TI) DLP4500 module, with 912 × 1140 micromirrors array in a diagonal pixel orientation. Additionally, custom resins were industrially tested with stereolithographic optical 3D printer Formlabs Form 2.

#### Nonlinear laser lithography

To perform nonlinear laser lithography (NLL) experiments custom setup was used: Pharos laser (515 nm, 300 fs, 200 kHz, Light Conversion Ltd), Scanlab HurryScan II Galvano-scanners, Aerotech positioning stages, Zeiss 20 × NA = 0.8 (transmittance 87%) and 63 × NA = 1.4 (transmittance 25%) objectives. A full description of the experimental setup can be found in a previous publication^27^. 3DPoli software was used to manage laser beam positioning inside the resin by controlling stages and scanners^32^.

#### Real-Time photorheometry

UV/VIS curing tests of AESO and its based photosensitized resins were carried out with a MCR302 rheometer from Anton Paar equipped with a plate/plate measuring system, as described in M. Lebedevaite *et al*. publication^43^. A Peltier-controlled temperature chamber with the glass plate (diameter of 38 mm) and the top plate PP08 (diameter of 8 mm) was used. The measuring gap was set to 0.3 mm. The samples were irradiated at room temperature by UV/VIS radiation in a wavelength range of (250–450) nm through the glass plate of the temperature chamber using a UV/VIS spot curing system OmniCure S2000, Lumen Dynamics Group Inc. The intensity of the irradiation was 9.3 W/cm^2^ (high pressure 200 W mercury vapour short arc). Shear mode with a frequency of 10 Hz and a strain of 0.3% was used. Storage modulus *G*’, loss modulus *G*”, and loss factor *tan δ* (*tan δ* = *G*”/*G*’) were recorded as a function of irradiation time. The onset of UV/VIS irradiation was at 60 s after the experiment start for all samples.

#### Sample characterization

Scanning electron microscope (SEM) Hitachi TM-1000 and Thermo Fisher Scientific Prisma E, optical profilometer Sensofar PL*μ* 2300 were employed to characterize the samples produced via DLP and NLL lithography. For SEM analysis, samples were coated 20 nm thick layer of gold using 150RS rotary pumped coater from Quorum Technologies Ltd.

## Conclusions

In summary, we have demonstrated a multi-scale (up to 5 orders) optical 3D printing (O3DP) of bio-based AESO compound. First of all, optical and rheological characteristics of the AESO-based resins with diverse formulations were investigated and found to be comparable with the commercially available ones. Then employing Lambert-Beer model, light penetration depth *h*_*a*_ and critical exposure duration *T*_*c*_ parameters were evaluated for a specific O3DP table-top setup and were 255 *μ*m and 0.36 s, respectively. After that polymerization depth *z*_*p*_ was calculated and cm-scale objects were produced using digital light processing (DLP) lithography at practical throughput and repeatability. Layer thicknesses of the printed objects showed good compliance with the calculations. Secondly, AESO suitability for nonlinear laser lithography (NLL) was assessed by determining the fabrication window (FW), which was found to be 3 under following conditions: average laser power *P* = 0.4–1.2 mW (*I* = 1.2–3.7 TW/cm^2^), scanning velocity *v* = 2.5–10 mm/s and distance between adjacent linear scans *d*_*xy*_ = 0.25–1.5 *μ*m. Such parameters are technically feasible with common laser lithography setups and convenient for additive manufacturing for precise mm-scale prototyping. Furthermore, by altering the focusing conditions, fine-manufacturing of the periodic structures with feature sizes smaller than 1 *μ*m, was demonstrated, not limiting the production of bulky monolith objects. To sum up, the revealed advances will broaden the O3DP applications by opening the way for multi-scale manufacturing out of a single bio-based material non-depending on the specific employed equipment.

## Supplementary information

Supplementary Information.
