# Supplementary material for: A Bio-Based Resin for a Multi-Scale Optical 3D Printing
Source: Sci Rep. 2020 Jun 16;10:9758. doi: 10.1038/s41598-020-66618-1 (PMC7297778; doi:10.1038/s41598-020-66618-1)
Supplement: Supplementary file 1 — Supplementary Information. [file 41598_2020_66618_MOESM1_ESM.pdf]

A Bio-Based Resin for a Multi-Scale Optical 3DPrinting

Edvinas Skliutas[1], Migle Lebedevaite[2], Sigita Kasetaite[2], Sima Rekštytė[1], Saulius Lileikis[3], Jolita Ostrauskaite[2], and Mangirdas Malinauskas[1,*]

[1]Laser Research Center, Physics Faculty, Vilnius University, Sauletekio Ave. 10, Vilnius, LT-10223, Lithuania

[2]Department of Polymer Chemistry and Technology, Kaunas University of Technology, Radvilenu Rd. 19, LT-50254, Kaunas, Lithuania

[3]3D Creative Ltd., Mokslininku St. 2a, Vilnius, LT-08412, Lithuania

[*]mangirdas.malinauskas@ff.vu.lt

Supplementary document

S1 Material characterization

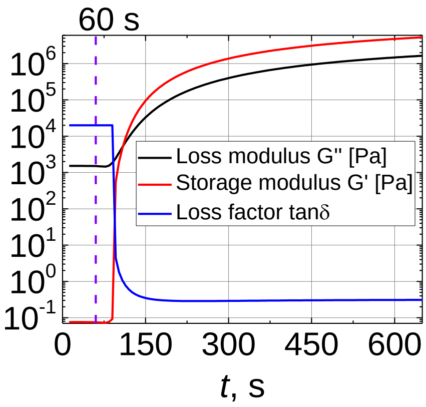

Figure S1.1. the dependencies of storage modulus *G'*, loss modulus *G"* and loss factor *tanδ* of AESO on irradiation time. The onset of irradiation (60 s) is marked with violet vertical dashed line [1].

Bio-renewable carbon (BRC) content is calculated according to the equation:

$$BRC = \frac{\text{Bio Sourced Carbon}}{\text{Bio Sourced Carbon} + \text{Fossil Carbon}} * 100\ \% \qquad \text{(S1.1)}$$

S2 Digital light processing lithography

S2.1 Optical 3D printer

Technical specification	Asiga Pico2 39 UV
Light source	385 nm UV LED
Irradiation intensity on resin tray window I_0	30 mW/cm^2
Resolution in XY plane	39 μm
Build size X, Y, Z	51×32×76 mm^3
Spot size for material testing	2 mm
Materials	III-rd party materials are available to use

Table S2.1. Digital light processing (DLP) optical 3D printer Asiga Pico2 39 UV technical specifications.

S2.2 Resin analysis for a digital light processing lithography

At first, optical absorbances ($A(\lambda)$) of the prepared resins were measured. For this, we used Shimadzu UVProbe spectrophotometer and 1 mm thick (s) cells filled with the resin. Then, device emission spectrum was measured using Avantes AvaSpec 2048 spectrometer. It allowed us to assess resins' absorption coefficients $\alpha(\lambda)$ at the wavelengths overlapping with the printers' emission spectra:

$$\alpha(\lambda) = \frac{A(\lambda)}{s} * \ln 10. \quad \text{(S2.1)}$$

Knowing an intensity distribution $I(\lambda)$ of the printers' light sources and using Beer-Lambert law based mathematical method, described in H. Gong's papers [2,3], we were able to evaluate energy dose (D), which can be absorbed by the resins

$$D(z,t) = t_{exp} \int_0^\infty I(\lambda) e^{-\alpha(\lambda) z} d\lambda, \quad \text{(S2.1)}$$

where t_{exp} stands for an exposure duration. Measuring the thickness of polymerized films z after various t_{exp} we calculated light penetration depth for a resin h_a^{exp} and a critical exposure duration T_c, required to reach the critical dose to start to polymerize the resin for an intensity $I(\lambda)$. h_a^{exp} value was determined from normalized exposure dose $D_n(z)$ calculations as a function of polymerization thickness z. z was measured with Sensofar PLμ 2300 profilometer and $D_n(z)$ was assessed by dividing absorbed energy dose from all energy dose illuminated a resin tray window (z = 0):

$$D_n(z) = \frac{D(z,t)}{D(0,t)} = \frac{\int_0^\infty I(\lambda) e^{-\alpha(\lambda) z} d\lambda}{\int_0^\infty I(\lambda) d\lambda}. \quad \text{(S2.2)}$$

Additional parameter a, which defines spectral overlap between resin absorbance and the source spectrum, was taken in to account. $D_n(z)$ can be approximated as

$$D_n(z) \approx a e^{-\frac{z}{h_a^{exp}}} + c = 1 - a\left(1 - e^{-\frac{z}{h_a^{exp}}}\right), \quad \text{(S2.3)}$$

where c = 1-a, when $D_n(0)$ = 1. T_c was evaluated from z dependence on t_{exp}, when h_a^{exp} and a values were fixed. Knowing all three parameters, we were able to calculate polymerization depth z_p when certain t_{exp} is applied:

$$z_p = h_a^{exp} \ln \frac{t_{exp} a}{T_c - t_{exp} + t_{exp} a}. \quad \text{(S2.4)}$$

Amount of PI	h_a^{exp}, [µm]	T_c, [s]
2 % w/w	180	0.15
1 % w/w	255	0.36
0.5 % w/w	440	0.51
0.25 % w/w	720	1

Table S2.2. Values of light penetration depth h_a^{exp} and critical duration T_c parameters' for acrylated epoxidizedsoy bean oil (AESO) based resins varying amount of the PI.

Applied exposure duration t_{exp}, [s]	Calculated polymerization depth z_p, [µm]	Average of measured thickness, [µm]
0.42	46	52±7
0.46	70	61±12
0.52	102	105±13
0.57	125	112±4
0.63	150	138±10
0.7	179	198±18

Table S2.3. Values of calculated polymerization depth z_p and printed monolayer membranes thickness for AESO based resins employing 385 nm wavelength irradiation exposure.

S2.3 Custom made resins testing under industrial optical 3D printing line

AESO was tested in "3D Creative" Ltd with with stereolithographic optical 3D printer Formlabs Form 2. "High temp" settings with 0.1 mm layer thickness were used for fabrication.

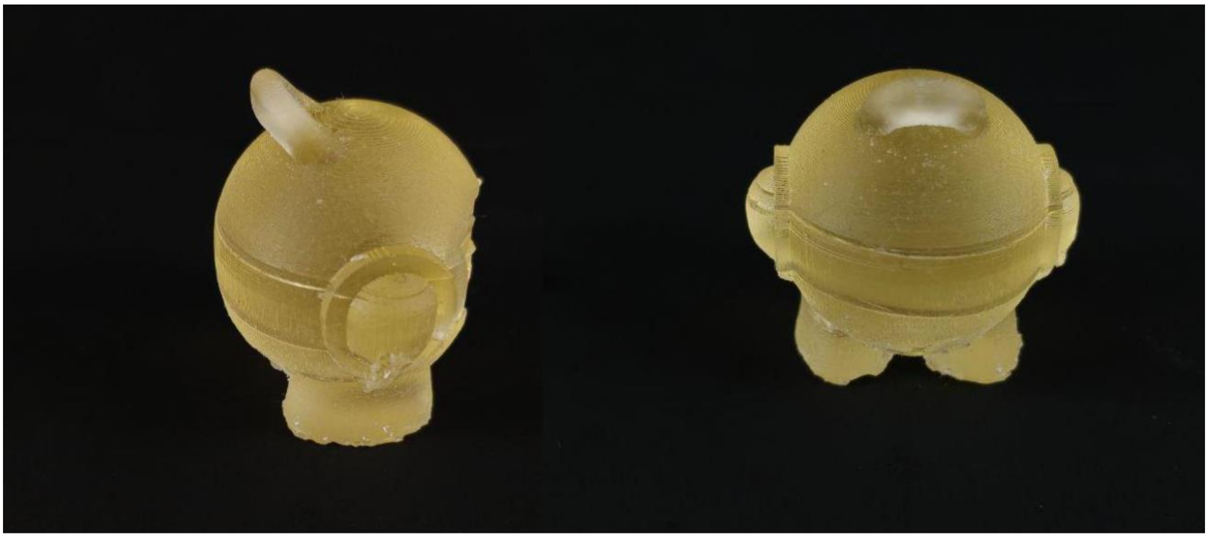

Figure S2.1. Optically 3D printed "Marvin" of size 24.5×19.5×25.4 mm^3 out of AESO bio-resin.

S3 Nonlinear laser lithography

S3.1 Resin analysis for a nonlinear laser lithography and fabrication parameters assessment

75×75 µm^2 size bi-layer scaffold structures, produced in AESO and AESO mixed with 1 % w/w of BAPO and diluted with ethyl lactate or Genomer 1122TF. Each log was 15 µm wide (*p*), distance between the logs (*d*) was set to 15 µm as well, resulting in total period of *T* = 30 µm. The layers were separated via vertical 20 or 30 µm height (*H*) columns.

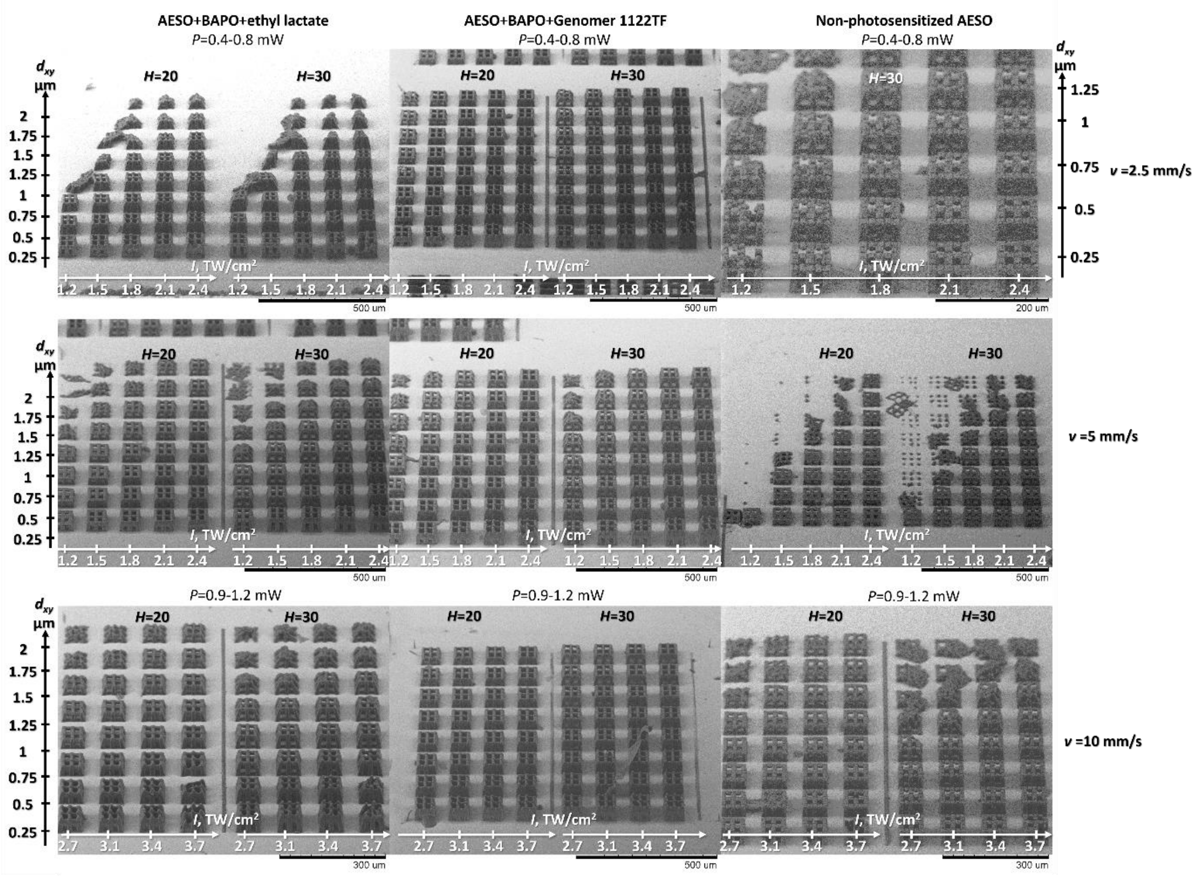

Figure S3.1. SEM images of arrays of 75×75 µm^2 manufactured scaffold structures. The structures were produced out of photosensitized AESO (1st column – AESO+BAPO+ethyl lactate, 2nd column – AESO+BAPO+Genomer 1122TF) and non-photosensitized AESO (3rd column). Applied average laser power *P* was 0.4-0.8 mW (1st and 2nd rows) and 0.9-1.2 mW. *P* was calculated to intensity *I* (white scales on the bottom of each image). Distance between adjacent beam scans d_{xy} is shown on black scales on the sides of images. Scanning velocity *v* was set to 2.5, 5 and 10 mm/s. *H* marks vertical column height.

S3.2 Spatial resolution determination

Nonlinear laser lithography (NLL) spatial resolution was determined in AESO, employing “Resolution bridges“ (RB) method. Depending on applied *P* and *v*, light intensity distribution changes at focal plane resulting in beams’ lateral (*D*) and longitudinal (*L*) sizes variation. This variation can be explained by Rayleigh model, according to which focused laser beam *D* and *L* dimensions can be expressed by such equations:

$$D = \frac{1.22\lambda}{NA}, \qquad \text{(S3.1)}$$

$$L = \frac{1.22n\lambda}{NA^2}. \qquad \text{(S3.2)}$$

n – refractive index, λ – wavelength, NA – objective numerical aperture. For more accurate estimation Gaussian intensity distribution at the focal plane must be taken into account:

$$I(r,z) = I_0 \frac{w_0^2}{w(z)^2} e^{-\frac{2r^2}{w(z)^2}} \quad \text{(S3.3)}$$

w_0 and I_0 are radius and intensity at beam waist. Beam radius can be expressed:

$$w(z) = w_0 \sqrt{1 + (\frac{z}{z_R})^2}. \quad \text{(S3.4)}$$

z_R is a Rayleigh length, which is the distance along the propagation direction of a beam from the waist to the place where the radius enlarges $\sqrt{2}$ times:

$$z_R = \frac{n w_0^2 \pi}{\lambda}. \quad \text{(S3.5)}$$

Multiphoton absorption is a probabilistic process. It occurs only when a certain light intensity (spatio-temporal photons density) level is applied. It is called threshold intensity I_{th}:

$$I_{th} = \frac{2PT}{f w_0^2 \pi \tau}. \quad \text{(S3.6)}$$

T – objective transmittance, f – pulse repetition rate, τ – pulse duration. Knowing I_{th}, D and L can be specified:

$$D(r) = w_0 \sqrt{\ln\left(\frac{I(r)}{I_{th}}\right)}, \quad \text{(S3.7)}$$

$$L(z) = 2z_R \sqrt{\sqrt{\frac{I(z)}{I_{th}}} - 1}. \quad \text{(S3.8)}$$

These equations determine dimensions of a volumetric pixel (voxel) at the focus plane and that it is the ellipsoid shape. Polymerization reaction is induced only in that voxel volume, where I equals or exceeds I_{th}. Increasing I optical damage threshold can be reached which starts gas bubbles formation. A gap between these two thresholds is called fabrication window (FW). Figure S3.2 (a) illustrates voxel and its dimensions D and L. Figure S3.2 (b) represents how D can be adjusted depending on applied I.

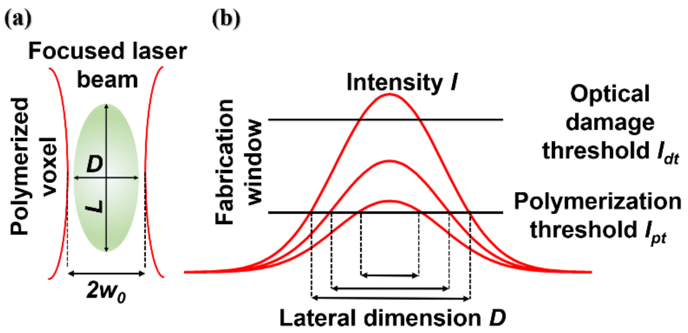

Figure S3.2. (a) – ellipsoid shape voxel (green) at the $2w_0$ diameter laser beam focal plane. D and L are lateral and longitudinal dimensions of the voxel. (b) – illustration, how D alters when different intensity I is applied. Fabrication window is defined by a lower black line (I_{pt}) showing intensity, at which polymerization reaction starts, and an upper one – an optical damage threshold (I_{dt}).

1. Lebedevaite, M., Ostrauskaite, J., Skliutas, E. & Malinauskas, M. Photoinitiator Free Resins Composed of Plant-Derived Monomers for the Optical µ-3D Printing of Thermosets. *Polymers,* **11**, 116 (2019).

2. Gong, H., Bickham, B. P., Woolley, A. T. & Nordin, G. P. Custom 3D printer and resin for 18 µm × 20 µm microfluidic flow channels. *Lab Chip* **17**, 2899–2909 (2017).

3. Gong, H., Beauchamp, M., Perry, S., Woolley, A. T. & Nordin, G. P. Optical approach to resin formulation for 3D printed microfluidic. *RSC Adv.* **5**, 106621 (2015).
